# Frequency Analysis of Failure Scenarios from Shale Gas Development

**DOI:** 10.3390/ijerph15050885

**Published:** 2018-04-29

**Authors:** Noura Abualfaraj, Patrick L. Gurian, Mira S. Olson

**Affiliations:** Department of Civil, Architectural, and Environmental Engineering, Drexel University, Philadelphia, PA 19104, USA; plg28@drexel.edu (P.L.G.); mso28@drexel.edu (M.S.O.)

**Keywords:** shale gas, fracking, exposure pathways, failure scenarios, occupational health risk

## Abstract

This study identified and prioritized potential failure scenarios for natural gas drilling operations through an elicitation of people who work in the industry. A list of twelve failure scenarios of concern was developed focusing on specific events that may occur during the shale gas extraction process involving an operational failure or a violation of regulations. Participants prioritized the twelve scenarios based on their potential impact on the health and welfare of the general public, potential impact on worker safety, how well safety guidelines protect against their occurrence, and how frequently they occur. Illegal dumping of flowback water, while rated as the least frequently occurring scenario, was considered the scenario least protected by safety controls and the one of most concern to the general public. In terms of worker safety, the highest concern came from improper or inadequate use of personal protective equipment (PPE). While safety guidelines appear to be highly protective regarding PPE usage, inadequate PPE is the most directly witnessed failure scenario. Spills of flowback water due to equipment failure are of concern both with regards to the welfare of the general public and worker safety as they occur more frequently than any other scenario examined in this study.

## 1. Introduction

The rapid growth of natural gas extraction in recent years, particularly from shale formations, has caused concern about the potential negative impact of this development on the surrounding environment, human health, and public safety. The increase in shale gas extraction is attributable to technological advancements, namely hydraulic fracturing and directional drilling, which have made natural gas extraction from low permeability shale formations feasible [1,2]. Hydraulic fracturing uses large volumes of pressurized water (2–5 million gallons of water for each fracturing operation) with chemical additives and a proppant (typically sand) to create fractures within the rock that allow trapped gas to diffuse to the well [1,3,4]. Meanwhile, horizontal drilling allows drilling into the shale layer, which runs horizontally and can lie at depths up to 8,000 ft. underground [1]. Because of this rapid growth, there have been many efforts to assess potential negative impacts in order to find ways to mitigate them and increase the safety of the natural gas industry [4,5,6,7,8,9,10,11].

Some potential sources of risk from this industry come from the hydraulic fracturing process as it produces wastewater (flowback water), which contains a portion of the water and additives that are initially pumped into the well along with dissolved constituents naturally occurring within the formation that are released immediately after the fracturing process and throughout the lifespan of the well [12,13,14,15]. Hydraulic fracturing wastewater generally has very high concentrations of salts and total dissolved solids (TDS), as well as levels of radionuclides, metals, and organic compounds that could be harmful to human health and pose treatment and disposal difficulties [16,17,18,19,20,21].

Another source of concern is from the failure to contain gases, causing methane to leak from wells into the subsurface or to flow to the surface causing blowouts, or from methane venting, which poses a risk to air quality [8]. There is also risk associated with transporting gas from the well pad to processing plants and compressor stations in surface pipelines [8,22].

To identify potential scenarios of concern regarding human health risks surrounding the natural gas drilling industry a survey of industry workers and regulators was developed and implemented to quantify the frequency of failure incidents and near-miss accidents at the wellhead site. The goal of this survey is to better understand scenarios of concern for human health risks as a result of operational failure incidents and regulatory violations during natural gas drilling in the Marcellus shale region in the state of Pennsylvania. The Marcellus region underlies two-thirds of the state Pennsylvania, and extends across the states of New York, Ohio, Maryland, and West Virginia with an estimated area of 54,000 square miles [12]. The scenarios investigated here involve a violation of regulations whether accidental or intentional focusing on recruiting subjects in the southwest and northeast regions of the state of Pennsylvania.

There have been many research efforts to identify the potential sources of exposure from the natural gas industry, particularly through water-related exposure pathways. In 2013, Resources for the Future implemented a survey of shale gas experts from government agencies, non-governmental agencies (NGOs), academia, and industry in order to identify high priority environmental risks from the shale gas industry [22]. The study asked 215 respondents to choose from 264 potential risk pathways as well as potential accidents from various stages of the development process in order to find a consensus and prioritize the pathways of exposure. The study identified 12 general pathways that were most frequently chosen by participants as being a priority. The pathways identified were classified based on whether they resulted in impacts to surface water, groundwater, air quality, or habitat disruption. While this study focused on general routine pathways, two accident risk pathways of concern were identified: cementing accidents and casing accidents.

In 2014, Vengosh et al. [23] conducted a review of risk to water resources from the development of shale gas. The study examines 4 different scenarios by which water resources can be negatively impacted from shale gas extraction: stray gas leaking into shallow aquifers, surface water contamination from spills and leaks, soil and river sediment contamination from wastewater, and the overuse of freshwater for hydraulic fracturing. While there is still a debate regarding the impact of shale gas development on groundwater, mostly due to the lack of baseline water quality data, it is recognized as a potential risk. Several studies [24,25] examined the effect of proximity to natural gas wells on methane contamination in surrounding drinking water wells in Pennsylvania. These studies found higher concentrations, on average, at locations that were within 1 kilometer of an active site. An isotopic analysis found evidence of thermogenic methane (methane formed at depths exceeding 1000 m under the surface at high temperatures) in active areas (within 1 kilometer of an active site) that was more prevalent in drinking wells further away from active natural gas sites. These studies have been criticized for limitations, such as their lack of baseline data, the lack of random sampling, the prevalence of methane in residential drinking wells in Pennsylvania regardless of proximity to natural gas wells, and the potential for confounding of geological characteristics with the presence of gas wells. A study by the United State Geological Survey conducted in 2013 found evidence of thermogenic methane in baseline drinking water samples predating natural gas activity in Sullivan County, Pennsylvania [26]. A recent study of 11,300 baseline dissolved methane analyses of drinking water samples in Pennsylvania provided by Chesapeake Energy found no statistically significant relationship between proximity to oil and gas wells and dissolved methane concentrations suggesting that the small sample sizes used in other studies may have been the reason behind the differences in results [27].

While these studies help identify the sources of concern that experts from various fields agree upon, they focus on environmental impacts without discussing human health. Bunch et al. [7] examines the effect of the shale gas industry on human health by looking at air quality in the Barnett shale region. The study examines data from several monitoring stations for volatile organic compounds (VOCs) in the region and compared them to federal and state health-based air standards to assess potential health effects. The study found that none of the VOCs examined posed any acute/short-term health risks, while one VOC (1,2-dibromoethane) had annual averages that exceeded chronic health risk standards. Bloomdahl et al., [5] examined health risks to workers due to inhalation of volatilized contaminants from holding ponds and concluded that these risks were minimal under typical exposure conditions.

Steinzor et al., [28] conducted a community survey of 108 individuals in the state of Pennsylvania to determine if any links existed between shale gas development and human health impacts. The study showed that the percentage of respondents reporting health symptoms frequently related to shale gas development, such as severe headaches, throat irritation, eye burning, and persistent cough, was higher for individuals who were less than 1500 feet away from the nearest drilling facility. The authors state that while the study does not definitively prove cause and effect, there appears to be a link between proximity to shale gas development sites and individuals reporting increased health effects. This indicates that these individuals are possibly being exposed to pollutants from the site that is negatively impacting their health.

An elicitation of health perceptions regarding unconventional shale gas development in the Marcellus region found that 22% of the 72 respondents perceived unconventional drilling as a health concern, while 42% attributed health symptoms to environmental factors, the most frequently selected of which was unconventional drilling activities [29].

Small et al., [30] provides an overview of risk associated with shale gas development while also identifying unresolved questions that require more knowledge. This assessment considers several domains including risk to water resources, air quality, climate change impacts, as well as public health impacts. While there is sufficient knowledge regarding surface water contamination, the effects on subsurface groundwater and soil are not as clear. Stray gases, spills, leaks, and accidental releases of wastewater are noted as potential risk to surface water and groundwater. The authors note, however, that there appears to be no systematic evidence of these types of failures as there are some uncertainties about the effects of the exposure pathways on human health. Workers and residents are vulnerable to dermal, inhalational, food, and water pathways, among others, from natural gas development. Identifying these pathways and quantifying their severity in order to determine risk is an important step in developing mitigation plans and strategies.

The goal of this study is to better understand industry perception towards the potential failure scenarios and exposure pathways that can occur on a shale gas development site through an elicitation of people who work in the natural gas industry. While other studies have assessed potential risk from general exposure pathways, this study examines specific failure events and elicited reports from industry participants on quantifiable accounts of actual past occurrences and near misses. As hydraulic fracturing has now been carried on for decades at thousands of wells, there is a considerable basis of professional experience for assessing both failures and near failure incidents. Following the approach of Galada et al., [31] this study draws on this knowledge to prioritize potential failure scenarios based on their potential impact on the health and welfare of the general public, potential impact on worker safety, how well safety guidelines protect against them, and how frequently they occur.

## 2. Materials and Methods

### 2.1. Survey Design

Scenarios of concern were identified through a literature review as well as through an interview with an oil and gas site field engineer. In general failure in this context is a failure of the required physical barriers separating the materials used in or derived from the extractions process from the environment and humans. Thus, the identification of failure scenarios consisted of identifying the barriers used and the times when such barriers could be breached. The field engineer described each step in the well development process (drilling, fracking, production and closure). At each step, the barriers used (liners, personal protective equipment, transport containers, etc.) and the locations where they were used were identified (well pad surface, subsurface, workers, etc.) were identified. Based on this information a set of scenarios consisting of particular barriers failing was drafted and reviewed by the field engineer. Those scenarios that were unrealistic based on field experience were eliminated. The remaining scenarios were modified as necessary to correspond with practice and terminology in the field. Based on this, the following list of twelve failure scenarios of concern was developed:

- Surface Pipe Failure
- Completion Failure in Subsurface (Liner/Casing Failure)
- Flowback Water Spills (due to Leaks in Equipment)
- Fracturing Fluid Spills (due to Leaks in Equipment)
- Surface Overflow of gases and/or fluids from Blowout
- Illegal Dumping of Flowback Water
- Impoundment Leakage or Spill
- Pit Leakage or Spill
- Transport Leakage or Spill of Flowback Water
- Transport Leakage or Spill of Fracturing Fluid
- Well Pad Liner Failure
- Improper or Inadequate Use of Personal Protective Equipment (PPE).

In addition, respondents were prompted to describe any additional scenarios of concern. 

The survey was created using SurveyMonkey Inc. (San Mateo, CA, USA). Potential subjects who were recruited to participate in the survey had the option of completing the survey over the phone or by following an email link to the SurveyMonkey website. Answers were recorded on SurveyMonkey for the duration of the study. After the data collection phase was complete, all data were downloaded and stored securely off-line. 

The survey consisted of 12 questions in 3 sections. The first section consisted of six questions and requested demographic information from each participant regarding their involvement in the oil and gas industry. After asking for each participant’s consent, the survey asked the participant’s job title, the number of years their work has involved shale gas, the approximate amount of time they spend on-site, the number of sites they’ve worked on over their career, and the region(s) in which they have worked in the shale gas field. The answers to these questions help give an idea of each respondent’s experience level.

The second part of the survey (questions 7 through 10) asked participants to rate the severity of each scenario, once in terms of potential negative impact to the general public, and once in regards to potential negative impact to on-site workers. In each of these questions, the survey has 6 answer options to choose from: Very Low, Low, Medium, High, Very High, and I Don’t Know. In the following question, participants are asked to rank the same 12 scenarios again, but this time in terms of how well they think safety guidelines protect against each failure. Participants had the same 6 answer options to choose from. In the last question in this section, participants were asked if any of the 12 scenarios had ever happened, whether they had first hand knowledge or second hand knowledge of the scenario occurring, if they scenario had almost happened but did not, if they had never witnessed the scenario happening, or if they thought it was impossible for the scenario to occur on-site. 

The last two questions of the survey were open-ended, one asking the participants to describe any other failure scenario related to shale gas and the other asking for suggestions for improvements to regulatory and safety practices that would make the shale gas industry safer. After completing all the questions, each participant was offered a gift card in the amount of $20 as compensation for their time.

### 2.2. Study Sample

A protocol for contacting participants was developed based on common practices for phone and email surveying techniques [32]. The protocol indicates when to make calls and send out emails, how long to wait before following up, how many times to follow up, and how to administer the survey over the phone. In general, it was recommended to wait four business days to follow up with a potential participant after establishing initial contact. If there is still no response four days after the first follow-up, the surveyor followed up one more time before considering the participant to be interested.

In cases where both an email address and phone number were available for the same contact, surveyors were directed to contact subjects by phone first. If there was no response after initial contact and two follow ups, the surveyor followed up one more time via email. Detailed records of each person contacted were kept in a database indicating each potential participant’s name, company, contact information, and response to the initial contact as well as each subsequent follow-up. In accordance with the study’s protocol for protection of human subjects, the information on whether or not a subject responded was not integrated with their responses to the survey.

The survey was targeted to individuals with experience in the oil and gas industry and knowledge of the operations at a natural gas site using a convenience sampling approach (as the goal was not to precisely quantify population frequencies of particular responses but rather to assess relative priorities of the scenarios). The desired sample size before distributing the survey was between 50 and 100 completed surveys. During the study time, 78 out of 231 people chose to participate in the survey. However, of those 78 respondents, only 60 people completed all the rating questions (1 through 10) and were included in the study database. 43 of the participants opted to receive the $20 gift card compensation that was offered.

### 2.3. Subject Enrollment

Subjects were enrolled through various methods:

- Professional Conferences: eligible participants were recruited at professional meetings and conferences related to the oil and gas industry. Possible participants were asked to complete the survey in person if they wished. Otherwise, a link to survey was sent via email to interested participants.
- Participant referrals: In some cases, participants would recruit other colleagues to participate in the survey.
- Linkedin InMail: InMail messages were sent to several professionals with experience in the shale gas industry through Linkedin Premium InMail messages.
- Professional Message Boards: A link to the survey was also posted on message boards for professional groups and associations, such as the ASCE-EWRI Collaborate message board, where other engineers and professionals in the water resources field were asked to participate and share the survey with any other professionals who may be interested.
- Shale gas industry directory: A directory of contacts and companies in the Marcellus and Utica Shale region purchased from HartEnergy. The directory contains contact information for over 6400 key personnel in the Marcellus-Utica region.

## 3. Results

The survey asked respondents to indicate their job titles within the oil and gas industry. Most of the participants provided an answer that fell within one of the categories presented in Table 1. In addition, the participants were asked about their level of experience in the natural gas industry. The average number of years that the survey participants have worked in shale gas development is 7.4 years, with 28 of the 78 participants having worked for 10 or more years in the industry. In addition, participants reported an average of 41% of their time working directly with shale gas activities on-site. However, 24 of the 78 respondents report spending less than 5% of their time dealing with on-site activities.

**Figure 1:**
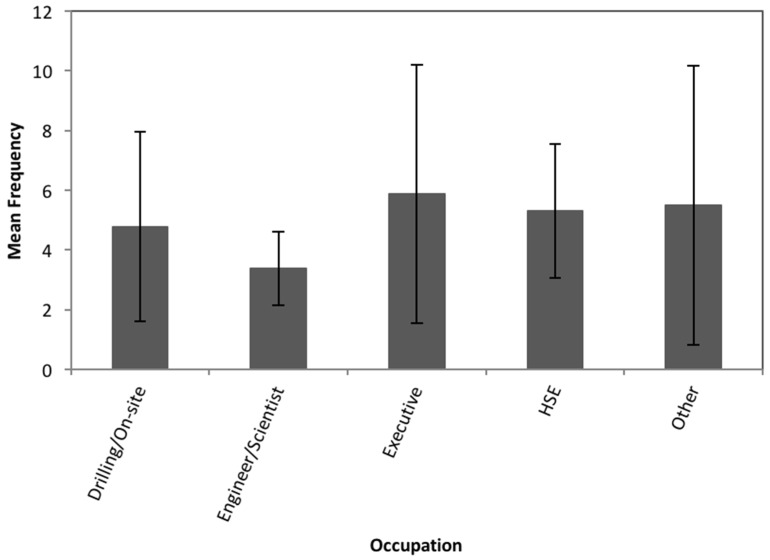
Average frequency of witnessing or learning of a scenario occurring by respondent occupation. Error bars represent the 95% confidence interval for the mean.

This information was used to determine whether or not there were any significant differences in the responses among the different occupations. The number of times that a scenario was reported to occur (whether based on first-hand or second-hand knowledge) was aggregated across all scenarios for each occupational category (Figure 1).

Participants whose job titles are in sales or public relations most frequently reported having knowledge of a failure scenario occurring. It should be noted that the sample size for this category is small, with only 3 responses. Executives and health and safety managers also had high averages of failure scenarios occurring, while engineers and scientists had the lowest rates of reported knowledge of any of the proposed scenarios happening (Figure 1). However, the confidence intervals for all categories overlap indicating that the differences are not statistically significant. This is confirmed by a Chi-squared test comparing expected and observed responses based on occupation to determine statistical significance at the 95% level (*p* = 0.05) that revealed no statistical significance.

The frequency with which subjects reported knowledge of scenarios occurring was correlated with both their number of years of experience and the percent of time they spent on site. No significant correlations were found. Correlating the respondents’ answers with their experience represented as a product of the number of years they have worked in the industry and the percentage of time spent on-site did not yield significant results either.

**Figure 2:**
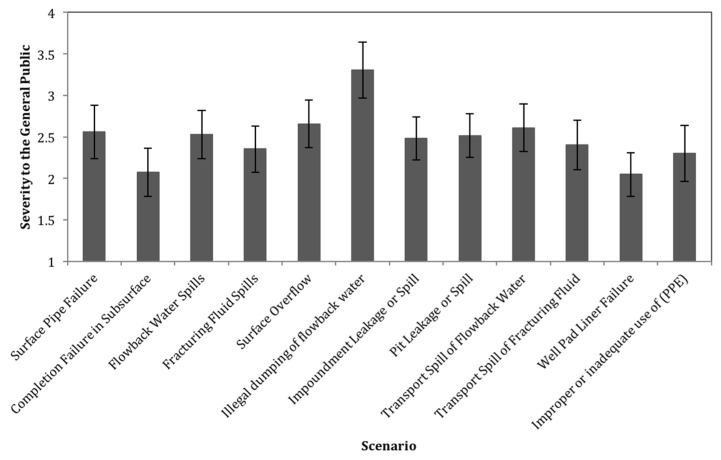
Average severity to the general public for each scenario (on a scale from 1 to 5). Error bars represent the 95% confidence interval for the mean.

The results of the second part of the survey were used to rank the severity of each scenario in terms of negative impact both to the general public and to the on-site workers. Figure 2 shows the result of question 7 of the survey where respondents were asked to rate the negative impact each scenario would have on the health and welfare of the general public if it were to occur. The results in Figure 2 are displayed on a scale from 1 to 5. In order to calculate the severity for each scenario, a number was assigned to each of the potential answer options on a Likert scale from 1 to 5 assuming equidistant values (Very Low = 1, Low = 2, Medium = 3, High = 4, Very High = 5). From the bar graph in Figure 2, the scenario of most concern in terms of potential negative impact to the general public is illegal dumping of flowback water. The confidence interval for this scenario does not overlap with any other scenarios, indicating that its rating is statistically significantly elevated compared to other scenarios. Well pad liner failures and completion failures have the lowest potential negative impact, but they are not statistically significantly different from the ratings of most other scenarios.

**Figure 3:**
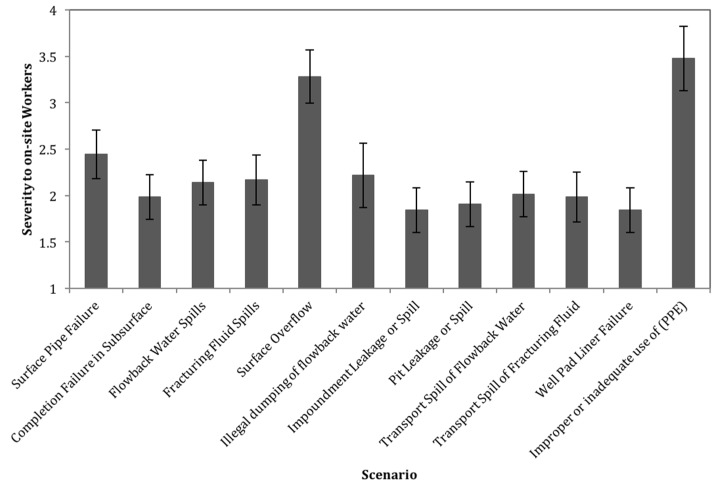
Average severity to on-site workers for each scenario (on a scale from 1 to 5). Error bars represent the 95% confidence interval for the mean.

The results for severity of risk to workers are shown in Figure 3. Based on the results of this survey, the most concerning scenario for worker safety is improper or inadequate use of personal protective equipment (PPE), followed closely by surface overflows of gases or fluids from a blowout, while the other scenarios have relatively similar severity scores. Ratings for failure to use PPE and risk from a blowout are not statistically distinct from each other, but are significantly elevated relative to all other scenarios.

**Figure 4:**
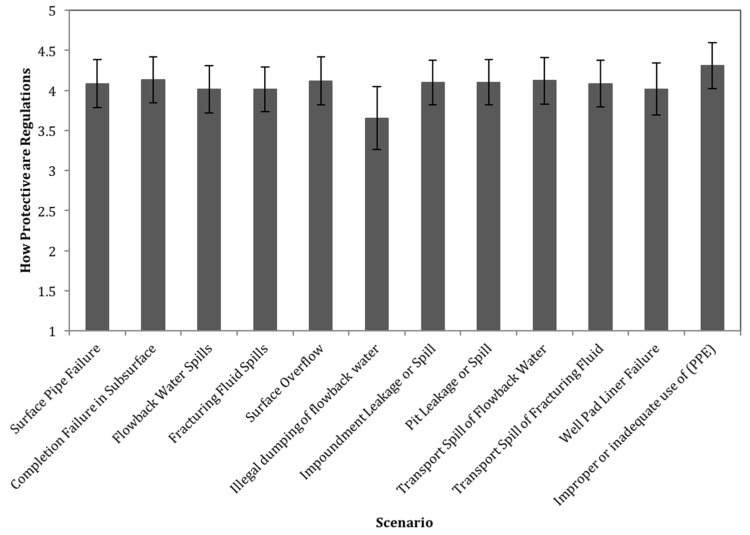
How protective current safety controls are against each scenario (on a scale from 1 to 5). Error bars represent the 95% confidence interval for the mean.

The results of question 9, which asked respondents to rate each scenario in terms of how well safety guidelines protect against the failure, are also displayed as a score from 1 to 5 (Figure 4). This was calculated similarly to the previous questions, where a number was assigned to each of the potential answers (Not at All = 1, Slightly = 2, Moderately = 3, Well = 4, Very Well = 5). Most scenarios had a score between 4.0 and 4.3, indicating that most respondents thought that these scenarios are moderately or well protected by existing safety regulations and guidelines. Illegal dumping of flowback water had the lowest average rating of 3.7, but this was not statistically significantly lower than the other average ratings.

**Figure 5:**
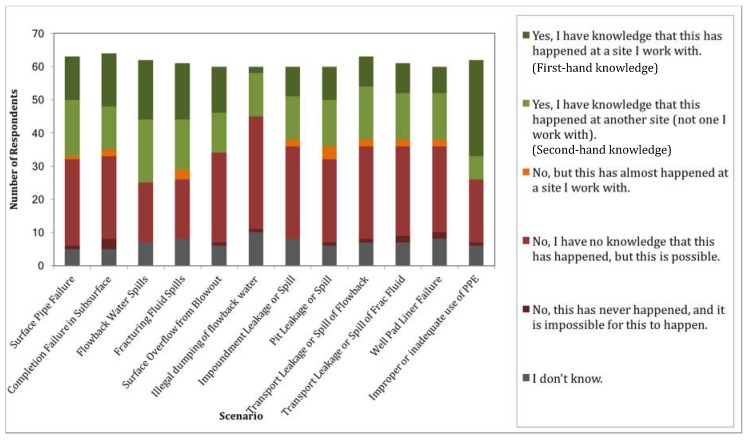
Frequency of “yes”, “no”, and “near miss” answers for each scenario.

The next question in the survey, question 10, asked if the scenario had ever happened and if they had direct knowledge of it occurring (at a site they worked with) or second-hand knowledge (at another site). They were also asked to indicate if the scenario had ever almost happened but did not, if it had never happened but was possible to occur, or if they thought the scenario was impossible. The results of this question are shown in Figure 5. All twelve scenarios were reported to have happened based on first-hand knowledge by more than one respondent. The scenario witnessed the least frequently was illegal dumping of flowback water with only 2 accounts of this being witnessed first-hand. Very few participants believed that any of the scenarios were impossible to occur. There are also few scenarios that have almost happened, with cases regarding fluid leaks and spills having the highest rates of near-misses. Illegal dumping of flowback water, while rated with the highest severity in terms of negative impact to the general public, has the lowest frequency of occurrence. While flowback water spills due to equipment failure have the highest overall (first-hand and second-hand) frequency of occurring, improper use of PPE has the highest frequency of being witnessed first-hand by survey participants. This is even more concerning since this scenario has the highest potential negative impact to on-site workers based on the responses to question 8.

**Figure 6:**
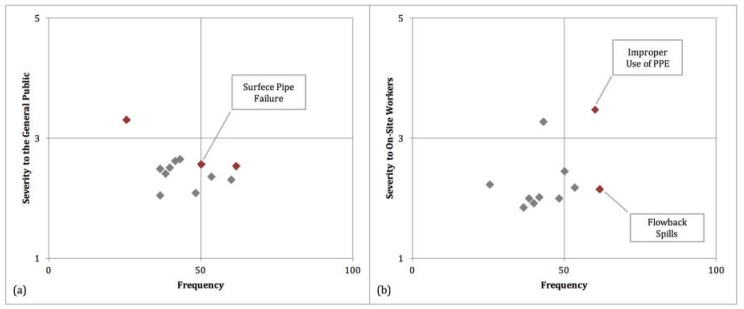
(**a**) Severity to the general public vs. Frequency for each scenario. (**b**) Severity to On-Site workers vs. Frequency for each scenario. Red markers indicate the scenarios that are non-dominated.

From there, the severity for each scenario was plotted against the frequency (first-hand and second-hand combined) in order to determine which scenarios were of highest priority in both categories. While this study cannot determine what the appropriate tradeoff is between the two categories, it can identify scenarios that are dominated by other scenarios (those that are lower than other scenarios on both attributes). This is presented in Figure 6, where Figure 6(a) shows the severity of risk to the general public against the frequency, and Figure 6(b) shows the same for severity to on-site workers. In both figures, the non-dominated scenarios are indicated in red. While it occurs the least frequently, illegal dumping of flowback water raises the most concern in terms of the health and welfare of the general public. In terms of worker safety, the highest concern comes from improper or inadequate use of personal protective equipment.

Spills of flowback water due to equipment failure are of concern both with regards to the welfare of the general public and worker safety. While these types of spills seem to have a low to moderate impact, the responses of this survey indicate that they occur more frequently than any other scenario when considering both first-hand and second-hand knowledge.

When prompted to describe any failure events that they witnessed first-hand, only two comments described a failure that was not addressed by this survey: a blowout due to collision with an existing well by a drilling bit (improper drilling bit steering), and wastewater release due to improper Stormwater management. Other responses to this question included incidents such as minor spills and leaks of fracturing fluid and produced water, failures during transportation, pit and impoundment leakages, and cement failures, all of which are included in the twelve proposed scenarios indicating that these scenarios are a plausible representation of actual conditions. 

## 4. Discussion

The scenarios developed here all merit attention as all were reported to have occurred by more than one respondent. Considering the demographics of the survey participants regarding occupation or work experience did not reveal any statistically significant relationships. However, additional analysis with a larger sample size, particularly with participants that spend a larger percentage of their on-site, could have differing results. While the scenarios presented here cannot be considered completely comprehensive, there were few additional scenarios identified by survey respondents. When prompted to describe any failure events that they witnessed first-hand, only two comments described a failure that was not addressed by this survey: a blowout due to collision with an existing well by a drilling bit (improper drilling bit steering), and wastewater release due to improper stormwater management. Other responses to this question included incidents such as minor spills and leaks of fracturing fluid and produced water, failures during transportation, pit and impoundment leakages, and cement failures, all of which are included in the twelve proposed scenarios. Improper drilling bit steering and improper stormwater management would probably merit inclusion in future studies of scenarios of concern. Nevertheless, the scenarios identified here appear to encompass a substantial set of plausible failures.

Illegal dumping of flowback water, while rated as the least frequently occurring scenario, is also the least protected by safety controls. The treatment and disposal of flowback and hydraulic fracturing wastewater falls under regulation at both the state and federal levels. However, based on the responses to this survey, there may be a need for better monitoring and accountability to ensure that it is not being disposed of improperly. While it seems to occur infrequently—only 13 respondents reported knowledge of this scenario occurring, 2 of which reported first-hand knowledge of this occurring at a site they worked with—improper disposal of wastewater that is often high in salinity, TDS, and radionuclides, can have negative impacts on public health and the surrounding environment. In addition, incidents regarding accidental spills and leaks of wastewater appear to occur relatively more frequently based on the results of this survey (Figure 5). While these events may be less severe in their impact, they are still a cause of concern for public health and safety.

Based on the results of question 9 (Figure 4), the general consensus among survey participants was that safety regulations and guidelines are adequately protective. However, there is an indication that some guidelines, particularly regarding proper protective equipment, are often ignored. This seeming contradiction may point to a need to revise not only the standard for protective equipment but the monitoring and compliance system. Several responses to the final two questions, which asked participants to describe any other failure scenario related to shale gas and to suggest any improvements to regulatory and safety practices, note that failures generally only occur when health and safety guidelines are violated by accident or ignored from negligence. Some notable suggested regulatory and safety improvements include more frequent inspections, more rigorous training requirements particularly for truck drivers carrying hazardous waste, increasing fines for illegal dumping of flowback water, and better enforcement of PPE usage on-site. This discrepancy highlights the level of uncertainty associated with hydraulic fracturing and shale gas development is reflective of the literature, which has also highlighted discrepancies between public and industry perceptions towards human health and environmental risk from shale gas activities [22,28], emphasizing the need for continued research efforts to evaluate and improve current practices and guidelines. In particular, efforts should be aimed at mitigating sources of human error or negligence regarding proper use of personal protective equipment by on-site workers. 

## 5. Conclusions

The twelve scenarios rated by participants of the survey gives insight into the natural gas industry’s perception of its own safety with regards to human health and worker safety. The results of the study revealed that illegal dumping of flowback water, while rated as the least frequently occurring scenario, is of concern to the industry as it is the least scenario protected by safety controls and could result in negative impacts to the general public. In terms of worker safety, the highest concern came from improper or inadequate use of personal protective equipment. While safety guidelines appear to be highly protective regarding PPE usage, inadequate PPE is the most reportedly observed failure scenario. Effort should be taken towards stronger enforcement of personal protection safety guidelines or otherwise protective of human error and negligence. Spills of flowback water due to equipment failure are of concern both with regards to the welfare of the general public and worker safety as they occur more frequently than any other scenario examined in this study.

## Figures and Tables

**Table 1:** Demographic occupational information for survey respondents.

Occupation	Number of Responses
Executive	7
Transportation	1
Health and Safety	16
Engineer/Scientist	32
Drilling/On-site	9
Public Relations/Sales	3
Academia	2
Regulator	2
Admin	1
General	3
No Answer	2
Total	78
